# Serendipitous detection of invasive malaria vector *Anopheles stephensi* in Kisumu, Kenya in June 2022

**DOI:** 10.1038/s41598-026-50986-1

**Published:** 2026-05-04

**Authors:** Bryson Alberto Ndenga, Kevin Omondi Owuor, Sammy Wambua, Brian Bartilol, Marta Maia, Joseph Mwangangi, Rodney Omukuti, Salome Chemutai, Daniel Arabu, Irene Miringu, Carren Bosire, Kavinya Mwendwa, Christabel Achieng Winter, Martin Kibet Rono, Francis Maluki Mutuku, Roz Taylor, Donal Bisanzio, Angelle Desiree LaBeaud, Keli Nicole Gerken

**Affiliations:** 1 Centre for Global Health Research, Kenya Medical Research Institute, Kisumu, Kenya; 2 Pwani University Biosciences Research Centre (PUBReC), Pwani University, Kilifi, Kenya; 3 Department of Biological Sciences (DBS), Pwani University, Kilifi, Kenya; 4 Research and Conservation Support Society (RECOURSE), Kilifi, Kenya; 5 School of Biodiversity One Health & Veterinary Medicine (SBOHVM), University of Glasgow, Glasgow, UK; 6 Centre for Geographic Medicine Research (Coast), Kenya Medical Research Institute - Wellcome Trust Research Programme, Kilifi, Kenya; 7 Centre for Global Health and Tropical Medicine, Nuffield Department of Medicine, University of Oxford, Oxford, UK; 8 Department of Chemistry and Biological Sciences, Technical University of Mombasa, Mombasa, Kenya; 9 Department of Public Health, School of Medicine and Health Sciences, Technical University of Mombasa, Mombasa, Kenya; 10 Department of Disease Control, London School of Hygiene and Tropical Medicine, London,, WC1E 7HT UK; 11 International Development Group, Global Health Division, RTI International, Washington, DC USA; 12 Department of Veterinary Science, University of Turin, Turin, Italy; 13 Department of Pediatrics, Division of Infectious Diseases, Stanford University School of Medicine, Stanford, USA; 14 Institute of Infection, Veterinary, and Ecological Sciences, University of Liverpool, Liverpool, UK; 15 International Livestock Research Institute, Nairobi, Kenya; 16 Infectious Diseases Epidemiology, Wageningen University and Research, Wageningen, The Netherlands

**Keywords:** *Anopheles stephensi*, Serendipitous, Kisumu, Kenya, Diseases, Ecology, Ecology, Genetics, Microbiology, Zoology

## Abstract

*Anopheles stephensi* is an invasive malaria vector that has been rapidly spreading within Africa since it was first identified in Djibouti in 2012. As of 2025, it has been identified in eight other countries: Ethiopia, Sudan, Somaliland, Eritrea, Nigeria, Kenya, Ghana and Niger. We collected mosquitoes from an urban slaughterhouse in Kisumu City in western Kenya in mid-2022 for bloodmeal analyses. 19.6% (11/56) of the amplicon sequence variants (ASVs) assigned using the basic local alignment search tool (BLAST) returned matches for mosquito species, one of which was *An. stephensi*, from one pool of five mosquitoes that had been morphologically classified as *An. gambiae s.l.* Further analysis of the mitochondrial cytochrome c oxidase 1 (*cox1*) gene later confirmed the presence of *An. stephensi*, which were distributed across two well-supported clades. Some haplotypes grouped with others from northern Kenya (Wajir and Marsabit), while others clustered with haplotypes from Ethiopia and Sudan. This serendipitous detection of *An. stephensi* is the first report of the species South of the Equator. Therefore, we recommend continued entomological and malaria case surveillance in this area to evaluate how shifting vector dynamics and vector-livestock interactions may impact future control initiatives.

**Supplementary Information:**

The online version contains supplementary material available at 10.1038/s41598-026-50986-1.

## Introduction

*Anopheles stephensi* is native to several countries in Asia^1,2^. It is known to contribute to the transmission of *Plasmodium vivax* and *P. falciparum*, including outbreaks in India’s cities and large towns, where it is the principal vector of urban malaria^3^. The presence of *An. stephensi* in Yemen was confirmed in 2021 in Aden City^4^, located in the southern part of the country, and this new range may have supported onward spread across the Horn of Africa, a scenario supported by genetic sequence analyses^5,6^. In Africa, *An. stephensi* was first identified in Djibouti in 2012 where it was associated with unusual *P. falciparum* malaria outbreaks in urban areas^7^. Since then, it has been reported in Ethiopia^8–11^, Sudan^12–14^, Somaliland^15^, Nigeria^16^, Kenya^17,18^, Ghana^19^, Eritrea^20^ and Niger Republic^21^.

The spread of *An. stephensi* in Africa threatens to reshape malaria transmission dynamics as we understand it, due to crucial differences between this invasive species and native malaria vectors. Firstly, while the primary malaria vectors native to Africa, *An. gambiae s.l.* and *An. funestus s.l.*, are found in rural areas, in temporary and large and more or less permanent natural habitats filled by rainwater, respectively, *An. stephensi* is also capable of breeding in man-made aquatic habitats such as water storage containers, cement cisterns, and construction site pits and ponds^5,17,18,20,22^. This ability makes it well-suited to urban environments, unlike *An. gambiae s.l.* and *An. funestus s.l.*, increasing the risk of malaria transmission in cities and towns where it was previously low. Secondly, *An. stephensi* adults not only bite indoors and at night, but also during the day and outdoors, and rest outdoors and in animal shelters^23,24^. These behaviours could decrease the effectiveness of mainstay insecticide-treated nets (ITNs) and indoor residual spraying (IRS) interventions. Thirdly, *An. stephensi* populations, both in Asia and in Ethiopia, exhibit host plasticity and have been found to prefer feeding on animals, especially livestock, over humans, and rest in animal holding areas^25–30^, which raises the question of how movement and trade of livestock could affect its dispersal^31^.

*Anopheles stephensi* is morphologically identified using two characteristic features, which are three light bands on the maxillary palps and two light interruptions in the second main dark area of wing vein 1^4,17,21^. They breed mostly in man-made aquatic habitats which include used car tires, discarded plastic jerricans, runoff from community tanks, shallow pit dug in gold mining sites, cut out water tanks, plastic water storage tanks, cemented water tanks and manholes and seasonal river pans^5,17,18,20,22^. The larvae and adults co-exist with other mosquitoes in the same aquatic habitats and resting places, respectively^18,21^. *Anopheles stephensi* is thought to have three biotypes: the “type” form which is more urban and anthropophilic, relative to the other two forms, and is thus a principal vector of urban malaria; “*mysorensis*” which is mainly found in rural areas and is relatively more zoophilic, hence a poor malaria vector; and the “intermediate” which is generally semi- and peri-urban, with its relative role in malaria transmission remaining unclear^24^.

As with many invasive species worldwide, detections of *An. stephensi* in Africa, particularly initial detections in new countries, are often serendipitous or part of non-targeted activities. For example, the first report on the continent in Djibouti was the result of *Aedes* surveillance activities^7^, the recent detection in Niger occurred during a general study of mosquito biodiversity^21^, and Ochomo et al.^17^, report that *An. stephensi* was detected in Kenya during “routine surveillance”. Furthermore, *An. stephensi* specimens collected in new regions are often morphologically misidentified. Most entomological surveillance teams across Africa are highly experienced in the morphological identification of primary malaria vectors *An. gambiae s.l.*, *An. funestus s.l.* and a few secondary vectors like *An. coustani* using popular keys^32,33^. However, recognition of novel species often requires additional training and molecular confirmation as demonstrated by reports of *An. stephensi* specimen which had been initially morphologically misidentified as *An. gambiae*^34^. Ahmed et al.^13^ collected 21 unknown *Anopheles* mosquitoes from Tuti Island in Khartoum, the capital city of Sudan, in 2018 two of which were morphologically identified and confirmed molecularly to be *An. stephensi*. Furthermore, while investigating insecticide resistance in *An. arabiensis* between 2016 and 2018, Ahmed et al.^12^ initially morphologically identified 149 specimens as *An. gambiae s.l.* that were found to be *An. stephensi* after further molecular analyses. In Kenya, Ochomo et al.^17^ morphologically misidentified two mosquitoes as *An. gambiae s.l.* that were later confirmed as *An. stephensi* by PCR. In our study, we present a serendipitous finding of *An. stephensi* from a pool of mosquitoes that were collected from the cattle holding pen at a large tertiary slaughterhouse in urban Kisumu City in western Kenya.

## Results

### Initial identification

In all, 248 amplicon sequence variants (ASVs) were inferred, 192 of which were classified to 14 vertebrate species (Mammalia and Aves) by exact matching against MIDORI2 reference database. Other than vertebrates, 19.6% (11/56) of the ASVs assigned using Basic Local Alignment Search Tool (BLAST) returned matches for mosquito species one of which was *An. stephensi* with a total of 15 hits (89–93% identity, 67–85% coverage, E-value < 1e-46 (Supplementary Table 1). Reads matching *An. stephensi* were traced to a pool of five (5) mosquitoes that had been morphologically classified as *An. gambiae* in the field.

### Oxford nanopore sequencing results

For the Oxford Nanopore Technologies (ONT) run, all the samples had over 40,000 reads after adaptor trimming (Table 1). Subsequent filtering for species-specific *An. stephensi* sequences, BLAST analysis confirmed the *An. stephensi* reads in four pools (Ksm001, Ksm043, Ksm049 and Ksm062), where they constituted less than 1% of the total reads, except one pool (Ksm015). The confirmed reads were mapped to *An. stephensi cox1* reference gene, and the resulting sequences error corrected using Medaka. The process generated four high-quality sequences which have been deposited in GenBank accession numbers: PX482732; PX482733; PX482734 and PX482735 (Table 1).

**Table 1:** Sample details, DNA concentration as measured by nanodrop and the number of reads obtained from the Oxford Nanopore Sequencing and those detected as specific for *Anopheles stephensi*.

Gene	Sample_ID	Accession No	DNA concentration	Total reads	*An. stephensi* reads
cox1	Ksm001	PX482733	28.4 ng/µL	101,337	4
cox1	Ksm043	PX482732	22.6 ng/µL	48,414	3
cox1	Ksm049	PX482734	21.7 ng/µL	81,137	7
cox1	Ksm062	PX482735	17.3 ng/µL	94,092	5

### Cytochrome c oxidase 1 (*cox1*) gene phylogenetics

**Fig. 1:**
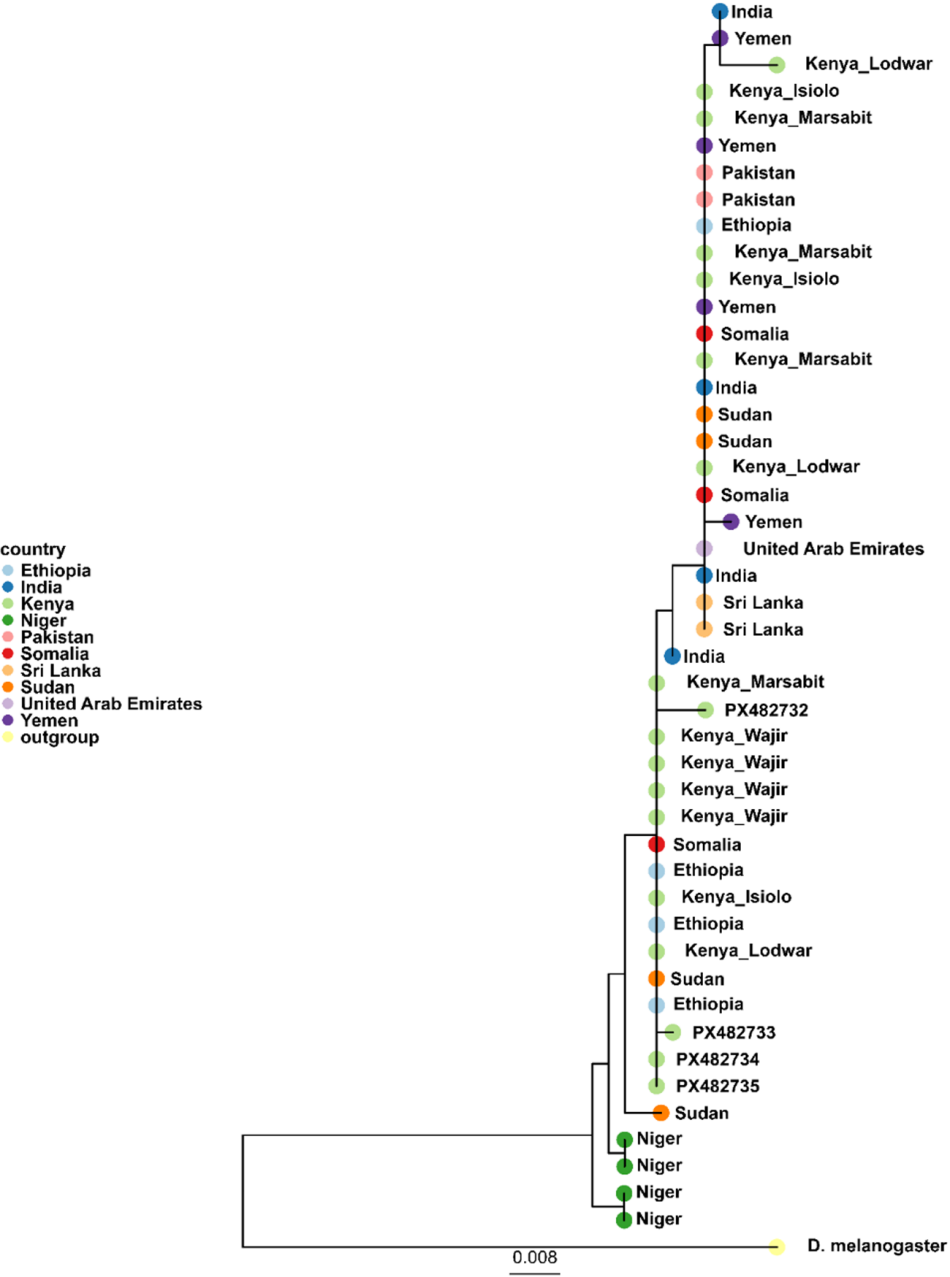
Phylogenetic tree of *Anopheles stephensi* mosquitoes collected in Kisumu City, Kenya, in June 2022 (PX482732; PX482733; PX482734 and PX482735) and reference sequences.

The mitochondrial *cox1* gene phylogeny showed that the Kisumu samples were distributed across two well-supported clades rather than a single group (Fig. 1). PX482732 grouped with haplotypes from northern Kenya (Wajir and Marsabit), while PX482733, PX482734 and PX482735 clustered with those from Ethiopia and Sudan (Fig. 1; Supplementary Table 2). The short branch lengths (0.008), are consistent with the limited genetic divergence of the *cox1* gene.

## Discussion

Our study reports the earliest known detection of *An. stephensi* south of the Equator identified in Kisumu, which is a major city and trade hub in western Kenya. Given year-round malaria transmission in Kisumu^35^, the detection of *An. stephensi* raises substantial public health concern. Since being first identified in Djibouti 13 years ago^7^, *An. stephensi* has been reported in both urban and rural areas in eight other countries in Africa^8,12,15–17,19–21^. It has shown itself capable of breeding in both artificial and natural habitats^8,17,18^ and resting indoor together with other mosquito species^21^. The city of Kisumu offers a wide variety of aquatic habitats that have been reported which could harbour *An. stephensi*. They include water collections at construction sites, mining sites, carwashes and animal watering points, plastic containers, water storage tanks, old car tires and seasonal river pans^10,17,18^. This implies that *An. stephensi* has ample opportunities of quickly establishing itself in this area. Further, livestock markets linking Kisumu to northern areas where *An. stephensi* is established could lead to further importations of the vector during transport, with animal holding areas providing a variety of suitable breeding and resting sites as reported in this study.

The presence of two genetically distinct lineages in Kisumu suggests at least two independent introductions, thus far highlighting the complex invasion dynamics of this species complex in Kenya. The most recent detection in Niger was linked to Ethiopia and Somaliland sequences, which would also suggest a role for long-distance transport along highways and roads^21^. Phylogenetic analyses also suggest cross-border spread between other countries in the Horn of Africa and multiple introductions from neighbouring countries. For instance, *An. stephensi* in Kenya from different regions had some haplotypes that were also present in sequences from Djibouti, Somalia and Ethiopia, but some other haplotypes found in Kenyan populations were only found in Djibouti and Northeast Ethiopia^18^. The phylogenetic placement of one sample (PX482732) with northern Kenyan populations implies movement along transport corridors linking the North and the West, in line with previous observations^17,18^. This could be the result of movement of livestock and livestock-keepers from northern regions, such as Turkana, into supplying markets in western Kenya for the Mamboleo slaughterhouse. Given the zoophilic tendencies of *An. stephensi* and its ability to breed in water stored in man-made containers, these journeys could transport the vector. In contrast, the clustering of the other samples (PX482733, PX482734 and PX482735) from Ethiopian and Sudanese lineages^12,26^ points to a separate introduction route from continental East Africa, perhaps associated with cross-border trade by highway over longer distances. These findings, along with the other detections across the continent^7,15,19,21,36^ indicate that the established presence of *An. stephensi* within Africa is dramatically underreported.

Serendipitous findings of *An. stephensi* in unexpected areas, as reported by Ahmed et al.^12,13^, suggest that the current distribution of this invasive malaria vector could extend far beyond the areas in which it has been formally documented thus far. This emphasizes the need for every entomological team working in Africa to screen samples for *An. stephensi* using both updated morphological and molecular identification tools, particularly given the possibility of misidentification. The initial morphological misidentification of *An. stephensi* vector as *An. gambiae* reported by Ochomo et al.^17^ and by us in this study underscores the need for updated training of field entomological teams accompanied by confirmatory molecular testing, to accurately identify this vector and map out its true distribution. Additionally, this study highlights the complications associated with molecular testing and interpretation of results from a sample that has been pooled for other reasons. We recommend that such samples be analyzed with an open-mind approach to increase the likelihood of detecting unanticipated yet important public health findings.

**Fig. 2:**
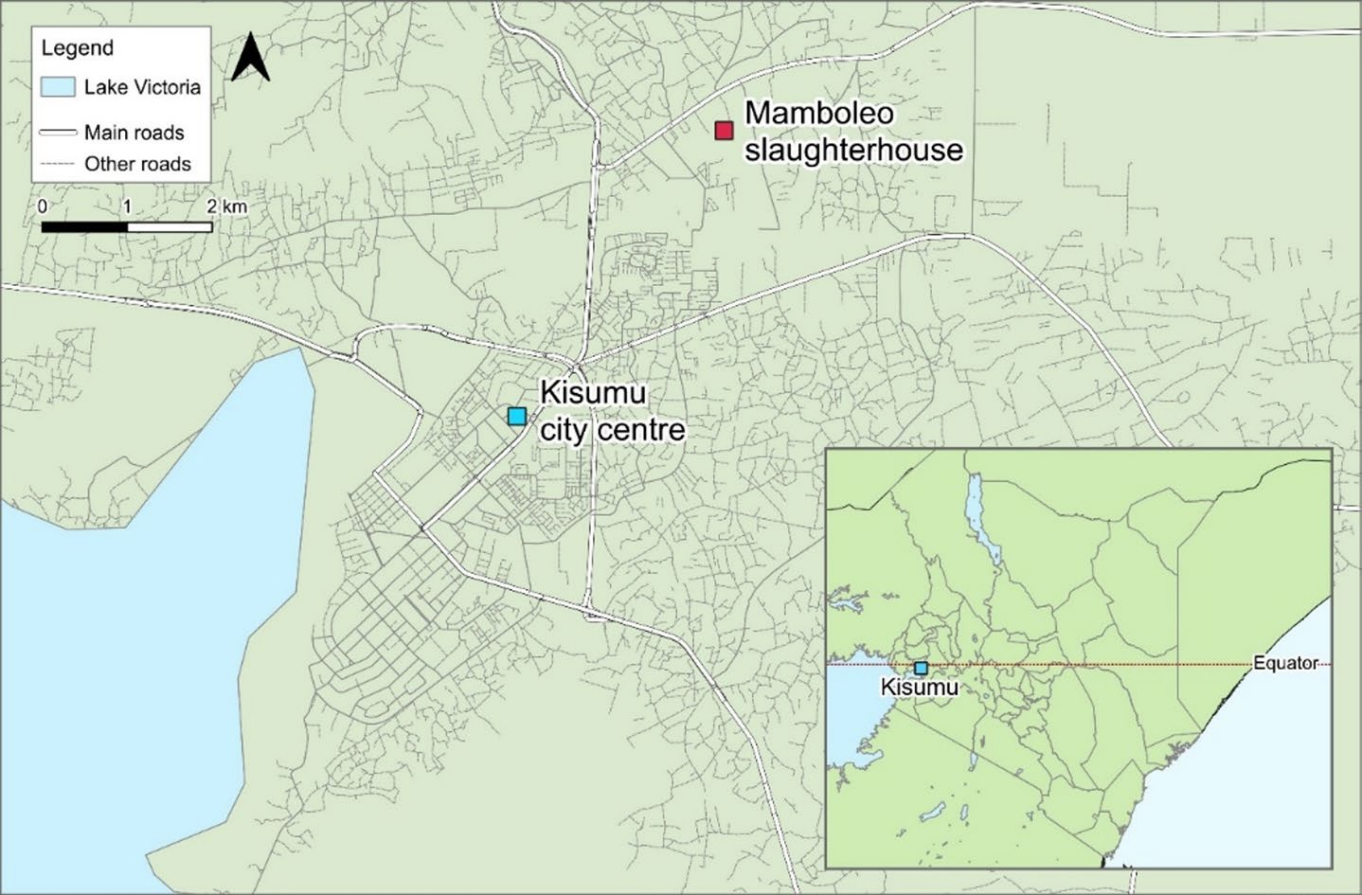
Study site map of Mamboleo slaughterhouse south of the Equator in Kisumu City, Kenya, where *Anopheles stephensi* was collected.

The detection of *An. stephensi* in Kisumu City adds another vector to the known list of native *Anopheles* in this area (*An. gambiae s.l.*, *An. funestus s.l.*, *An. coustani*, *An. pharoensis*, *An. maculipalipis*, and *An. leesoni*)^37^. The presence of this invasive malaria vector in this area could increase local circulation of *P. falciparum*^38–40^ and a possible introduction of *P. vivax* may potentially create a situation similar to that seen in Djibouti in 2013 and 2014^7^. Since the samples used in this study were collected over three years ago, *An. stephensi* may have already expanded its presence and could be actively contributing to local malaria transmission. This is especially probably, as our detection at the slaughterhouse was in a densely populated and well-connected area of the city (Fig. 2). This emphasizes an urgent need to conduct follow up studies in Kisumu City and the surrounding regions, particularly in Mamboleo.

Several limitations were encountered during this study. Firstly, the entomology team not trained in morphological identification of *An. stephensi* specifically, and misidentification could be avoided by providing such training and increasing awareness of the presence of *An. stephensi* within the African continent. Secondly, DNA extraction was carried out on pooled samples, mixing *An. gambiae* and *An. stephensi* DNA, which challenged the initial identification of *An. stephensi*, and *An. gambiae* sequences may have dominated BLAST searches, reducing the ability to note *An. stephensi* hits. Lastly, limited funds for molecular testing further delayed the confirmation of the presence of *An. stephensi*.

## Conclusion

This study reports the initial detection of the invasive malaria vector *An. stephensi* south of the Equator in an urban, malaria-endemic area within Kisumu City, western Kenya. A key takeaway from this serendipitous detection is the urgent need for increased awareness of *An. stephensi* across entomological teams in Africa, and rapid training of its morphological identification, to facilitate rapid reporting and response. The presence of *An. stephensi*, capable of transmitting both *P. falciparum* and *P. vivax*, poses a significant public health threat, and could considerably shift malaria transmission dynamics in endemic zones within the Lake Victoria region. Therefore, we recommend continued entomological surveillance, monitoring of malaria cases at this site and the entire malaria endemic area and evaluation of how shifting vector dynamics and vector-livestock interactions may impact future malaria control initiates. Since, people, livestock and *An. stephensi* mosquitoes were all found at the same slaughterhouse, there is possibility that their female adult mosquitoes obtained blood meals from both livestock and human beings as reported by Carter et al.^31^. In addition, Carter et al.^31^ categorically noted that there is no likelihood that livestock themselves transport the mosquitoes. However, livestock may attract these mosquitoes and the water containers they travel with may serve as mobile breeding habitats up to their destinations^31^. Given the slaughterhouse setting of this detection, and the status of Kisumu as a major trade hub, we also suggest an evaluation of the source(s) of livestock arriving at slaughterhouses, in order to understand how such movement and trade may be aiding in the dispersal of *An. stephensi*. In addition, as we try to understand its spread, this information highlights the importance of the One Health approach and inclusion of livestock movement studies^31,41^ to study more on dispersal of *An. stephensi* and can be used for developing targeted risk mitigating approaches. For example, we propose using livestock transport channels as targeted vector control points. Vehicles carrying livestock could be sprayed with insecticide before departure and upon arrival to prevent “hitchhiking” by this vector, whose spread to date has closely followed transport infrastructure.

## Methods

### Study site and sampling

In the context of a study investigating Rift Valley Fever, entomological collections were conducted at Mamboleo Slaughterhouse (0° 3’ 25.4’’ S, 34° 47’ 10.9’’ E, approximately 6.4 km South of the Equator) in Kisumu City, western Kenya (Fig. 2) as described by Gerken et al^42^.

In brief, we sampled mosquitoes using ovitraps, Biogents (BG) sentinel trap and a Prokopack aspirator used both indoors and outdoors from May 16^th^ 2022 to July 1^st^ 2022. Sampling was done anytime between 08:45 h and 01:30 h and lasted for one hour. We completed subsequent samplings biweekly. We killed the mosquitoes we collected using a brief pyrethrum aerosol spray before we morphologically identified them in a field station and recorded the data on a field form. We further sorted female mosquitoes according to their blood-feeding stages as unfed, blood-fed, half-gravid or gravid. We then preserved the mosquitoes in silica gel self-indicating 6-20mesh (Blue) − 500gm (Loba Chemie Pvt. Ltd, Jehangir Villa, 107, Wodehouse Road, Colaba, Mumbai 400 005, India) with the intention for further blood meal testing. A total of 16 blood-fed *An. gambiae* mosquitoes were batched into three sample vials, each containing five or six blood-fed individuals, were transported on dry ice to Pwani University Biosciences Research Centre (PUBReC) in Kilifi, Kenya, for determination of blood meal sources where initial detection of *An. stephensi* was made as described below.

### Initial identification

Genomic DNA was extracted from the mosquito abdomens using the TIANamp Genomic DNA Kit (Tiangen, Beijing, China). Quality and quantity of DNA was analyzed by NanoDrop 2000 C spectrophotometer (Thermo Scientific Inc, USA) and verified on 1% agarose gel electrophoresis. To identify blood meal sources, we PCR-amplified ~ 300 bp (bp) of the cytochrome b barcode^43^ which was high-throughput-sequenced, at Macrogen Inc., Seoul Korea, using the Illumina 300 × 2 bp platform (Illumina, USA). The amplicon sequence data were analyzed with the DADA2 (version 1.21.0) bioinformatics pipeline^44^ implemented in the R programming language (version 4.1.1). After quality preprocessing, high quality reads were denoised and clustered into unique sequences i.e., amplicon sequence variants (ASVs). We assigned taxonomy to the ASVs using two approaches. We assigned taxonomy, first, by exact ASV matching against DADA2-trained MIDORI2 reference database (version GB254) which includes eukaryotic mitochondrial sequences^45^. ASVs that were not assigned by this approach were compared against the NCBI database using BLAST^46^. Raw sequences are available in SRA database of NCBI under project accession number PRJNA966766.

These initial identifications of *An. stephensi* were deemed indicative however insufficient to make definitive conclusions on the species identity. Therefore, confirmatory testing using the cytochrome c oxidase subunit 1 (*cox1*) gene was done in collaboration with the Kenya Medical Research Institute-Wellcome Trust Research Programme in Kilifi as described below.

### Confirmatory identification

#### PCR analysis

For the detection of *An. funestus* and *An. gambiae*, primers developed by Koekemoer et al.^47^ and Scott et al.^48^, respectively, were used. The PCR mixture consisted of the 5 µL of 2X GoTaq master mix (cat. M7122, Promega, Wisconsin, US), 0.5µL of primers, 0.5 µ nuclease free water and 1.5 µL of the sample. The PCR conditions were 95 °C for 5 min (mins), 40 cycles of 95 °C for 15 s (secs), 48 °C for 20 s, and 72 °C for 45 s, with a final extension step at 72 for 10 min.

For *An. stephensi* we used primers developed by Djadid et al.^49^ and Balkew et al.^9^. The PCR mix consisted of 5 µL of 2X GoTaq master mix, 0.5µL of primers, 2.5 µL nuclease free water and 1.5 µL of the sample. The PCR conditions were 95 °C for 5 min (mins), 40 cycles of 95 °C for 15 s (secs), 55 °C for 20 s, and 72 °C for 45 s, and finally a final extension step at 72 °C for 10 min.

For the amplification of the cytochrome c oxidase 1 (*cox1*) gene, we used primers developed by Folmer et al.^50^. The PCR mix consisted of 5 µL of 2X GoTaq master mix, 0.5µL of primers, 2.5 µL nuclease free water and 1.5 µL of the sample. The PCR conditions were 95 °C for 5 min (mins), 40 cycles of 95 °C for 15 s (secs), 48 °C for 20 s, and 72 °C for 45 s, and finally a final extension step at 72 °C for 10 min.

#### Amplicon generation for sequencing

The amplicons from the *cox1* gene PCR were cleaned using the QIAquick PCR purification kit (cat. 28104, Qiagen, Hilden, Germany). The yield was then quantified using NanoDrop ND 1000 spectrophotometer (ThermoFisher Scientific, Massachusetts, USA).

#### Oxford nanopore sequencing

The amplicons were normalized to a total concentration of 100 nanograms (ng). Thereafter, libraries were prepared using the Native barcoding kit (SQK-NBD114.96) from the Oxford Nanopore Technologies (ONT) and sequencing was performed on the GridION platform using a R10.4.1 flow cell. The Dorado v7.3.9^51^ software was employed for base calling.

### Data analysis

Adaptor trimming was performed using Porechops v0.2.4^52^. to identify *An. stephensi* reads we used Seqkit v2.10.1 to search for species-specific DNA sequence (ACTATACTTCTAGAAATTAAAAGAG) in the raw FastQ files. Putative *An. stephensi* reads were confirmed by BLAST analysis against the NCBI’s nucleotide database (core_nt) using default parameters. Reads were then mapped to the *An. stephensi cox1* reference sequence using Minimap2 v2.30^53^ with subsequentBAM conversion and indexing via Samtools v1.19.2^54^. Finally, error correction and consensus sequence generation were performed using Medaka v2.1.1^55^.

Phylogenetic analysis included samples from Kisumu supplemented with sequences from GenBank. Multiple sequence alignment was performed using Muscle v5.3^56^, followed by trimming of overhanging regions in Aliview v1.28^57^. Maximum likelihood phylogenies inferred with IQ-TREE v2.3.4^58^ which used Bayesian Information Criterion to select Kimura 3-parameter (K3Pu + F) as the best substitution model as inferred by jModelTest. The analysis included 1000 bootstraps replicates. The tree was then visualized in R v4.2.3^59^ using the ggtree and ggplot2 packages.

## Electronic Supplementary Material

Below is the link to the electronic supplementary material.

Supplementary Material 1

## Data Availability

All sequences generated in this study have been deposited in NCBI GenBank under accession numbers: PX482732, PX482733, PX482734 and PX482735 for COXI.
