# Supplementary material for: Serendipitous detection of invasive malaria vector Anopheles stephensi in Kisumu, Kenya in June 2022
Source: Sci Rep. 2026 May 4;16:20517. doi: 10.1038/s41598-026-50986-1 (PMC13328431; doi:10.1038/s41598-026-50986-1)
Supplement: Supplementary file 1 — Supplementary Material 1 [file 41598_2026_50986_MOESM1_ESM.docx]

**Supplementary Tables**

**Supplementary Table 1:** Sample details of DNA concentration as measured by nanodrop and the number of reads obtained from the Oxford Nanopore Sequencing.

| **Gene** | **Sample_ID** | **DNA concentration** | **Number of reads** |
| --- | --- | --- | --- |
| cox1 | 1 | 28.4 ng/µL | 101,337 |
| cox1 | 15 | 21.6 ng/µL | 92,985 |
| cox1 | 43 | 22.6 ng/µL | 48,414 |
| cox1 | 49 | 21.7 ng/µL | 81,137 |
| cox1 | 62 | 17.3 ng/µL | 94,092 |
| cox2 | PC | 10.1 ng/µL | 234,484 |

**Supplementary Table 2:** GenBank accession numbers for reference sequences in relation to the *Anopheles stephensi* mosquitoes collected in Kisumu City, Kenya, in June 2022 (PX482732; PX482733; PX482734 and PX482735).

| Accession number | Country | Kenyan site | Sample ID |
| --- | --- | --- | --- |
| PX482732 | Kenya | Kisumu | Ksm043 |
| PX482735 | Kenya | Kisumu | Ksm062 |
| PP752284.1 | Yemen |  |  |
| ON421573.1 | Somalia |  |  |
| PP387837.1 | Yemen |  |  |
| PP752283.1 | Yemen |  |  |
| OQ847826.1 | India |  |  |
| OR607951.1 | Kenya | Lodwar |  |
| PQ529995.1 | Kenya | Isiolo |  |
| PQ529952.1 | Kenya | Marsabit |  |
| OM865140.1 | Yemen |  |  |
| KF406693.1 | Pakistan |  |  |
| KF406680.1 | Pakistan |  |  |
| OM801697.1 | Ethiopia |  |  |
| PQ529956.1 | Kenya | Marsabit |  |
| PQ529958.1 | Kenya | Isiolo |  |
| PQ529996.1 | Kenya | Marsabit |  |
| MT899149.1 | India |  |  |
| OK216687.1 | Sudan |  |  |
| OK216469.1 | Sudan |  |  |
| OR607949.1 | Kenya | Lodwar |  |
| ON421574.1 | Somalia |  |  |
| MK170098.1 | United Arab Emirates |  |  |
| MF124608.1 | Sri Lanka |  |  |
| MF124610.1 | Sri Lanka |  |  |
| MW549046.1 | India |  |  |
| PP469559.1 | India |  |  |
| NC_024511.2_1474-3009 | outgroup Drosophila melanogaster |  |  |
| PV200299.1 | Niger |  |  |
| PV200298.1 | Niger |  |  |
| PV200295.1 | Niger |  |  |
| PV200296.1 | Niger |  |  |
| OK216660.1 | Sudan |  |  |
| PX482734 | Kenya | Kisumu | Ksm049 |
| PX482733 | Kenya | Kisumu | Ksm001 |
| OM801698.1 | Ethiopia |  |  |
| OK216358.1 | Sudan |  |  |
| OR607950.1 | Kenya | Lodwar |  |
| OM801702.1 | Ethiopia |  |  |
| PQ529988.1 | Kenya | Isiolo |  |
| OK663481.1 | Ethiopia |  |  |
| ON421572.1 | Somalia |  |  |
| PQ529974.1 | Kenya | Wajir |  |
| PQ529949.1 | Kenya | Wajir |  |
| PQ529980.1 | Kenya | Wajir |  |
| PQ529982.1 | Kenya | Wajir |  |
| PQ529955.1 | Kenya | Marsabit |  |
